# Bis{*N*-methyl-*N*′-[1-(pyridin-2-yl)ethyl­idene]ethane-1,2-diamine}­zinc bis­(perchlorate)

**DOI:** 10.1107/S1600536811026079

**Published:** 2011-07-06

**Authors:** Chen-Yi Wang

**Affiliations:** a Department of Chemistry, Huzhou University, Huzhou 313000, People’s Republic of China

## Abstract

The title mononuclear zinc(II) complex, [Zn(C_10_H_15_N_3_)_2_](ClO_4_)_2_, was obtained by the reaction of 2-acetyl­pyridine, *N*-methyl­ethane-1,2-diamine and zinc perchlorate in methanol. The asymmetric unit of the complex contains two independent [Zn(C_10_H_15_N_3_)_2_]^2+^ cations and four perchlorate anions. The Zn^II^ atom in each complex cation is six-coordinated by two pyridine N, two imine N and two amine N atoms from two *N*-methyl-*N*′-[1-(pyridin-2-yl)ethyl­idene]ethane-1,2-diamine Schiff base ligands in a distorted octa­hedral geometry. The pyridine rings in each of the complex cations are approximately perpendicular to each other, making dihedral angles of 88.4 (3) and 87.9 (3)°. The perchlorate anions are linked to the complex cations through N—H⋯O hydrogen bonds

## Related literature

For Schiff base complexes we have reported previously, see: Wang (2009 ▶); Wang & Ye (2011 ▶). For other similar zinc complexes, see: Cai *et al.* (2009 ▶); Yang *et al.* (2009 ▶); Bing *et al.* (2010 ▶); Wang *et al.* (2010 ▶).
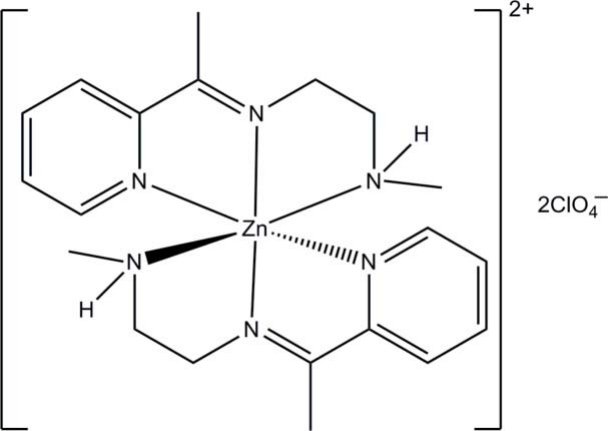


## Experimental

### 

#### Crystal data

[Zn(C_10_H_15_N_3_)_2_](ClO_4_)_2_
*M* *_r_* = 618.77
Monoclinic, 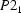

*a* = 12.7999 (3) Å
*b* = 15.8414 (4) Å
*c* = 13.6869 (3) Å
β = 102.461 (1)°
*V* = 2709.89 (11) Å^3^
*Z* = 4
Mo *K*α radiation
μ = 1.16 mm^−1^
*T* = 298 K
0.32 × 0.30 × 0.27 mm

#### Data collection

Bruker SMART CCD area-detector diffractometer
Absorption correction: multi-scan (*SADABS*; Sheldrick, 1996 ▶) *T*_min_ = 0.708, *T*_max_ = 0.745
12863 measured reflections
7055 independent reflections
5567 reflections with *I* > 2σ(*I*)
*R*_int_ = 0.034
θ_max_ = 23.9°

#### Refinement

*R*[*F* ^2^ > 2σ(*F* ^2^)] = 0.059
*wR*(*F* ^2^) = 0.168
*S* = 1.04
7055 reflections
675 parameters
7 restraints
H-atom parameters constrained
Δρ_max_ = 0.73 e Å^−3^
Δρ_min_ = −0.63 e Å^−3^
Absolute structure: Flack (1983 ▶), 2683 Friedel pairs
Flack parameter: 0.01 (2)

### 

Data collection: *SMART* (Bruker, 1998 ▶); cell refinement: *SAINT* (Bruker, 1998 ▶); data reduction: *SAINT*; program(s) used to solve structure: *SHELXS97* (Sheldrick, 2008 ▶); program(s) used to refine structure: *SHELXL97* (Sheldrick, 2008 ▶); molecular graphics: *SHELXTL* (Sheldrick, 2008 ▶); software used to prepare material for publication: *SHELXTL*.

## Supplementary Material

Crystal structure: contains datablock(s) global, I. DOI: 10.1107/S1600536811026079/sj5175sup1.cif

Structure factors: contains datablock(s) I. DOI: 10.1107/S1600536811026079/sj5175Isup2.hkl

Additional supplementary materials:  crystallographic information; 3D view; checkCIF report

## Figures and Tables

**Table 1:** Selected bond lengths (Å)

Zn1—N5	2.087 (6)
Zn1—N2	2.105 (6)
Zn1—N3	2.163 (6)
Zn1—N4	2.210 (7)
Zn1—N6	2.215 (7)
Zn1—N1	2.278 (6)
Zn2—N11	2.090 (7)
Zn2—N8	2.109 (7)
Zn2—N9	2.188 (7)
Zn2—N7	2.209 (6)
Zn2—N12	2.224 (7)
Zn2—N10	2.225 (7)

**Table 2:** Hydrogen-bond geometry (Å, °)

*D*—H⋯*A*	*D*—H	H⋯*A*	*D*⋯*A*	*D*—H⋯*A*
N12—H12*A*⋯O5^i^	0.91	2.55	3.425 (13)	163
N12—H12*A*⋯O7^i^	0.91	2.38	3.175 (17)	146
N9—H9*C*⋯O8^i^	0.91	2.45	3.315 (14)	158
N9—H9*C*⋯O5^i^	0.91	2.39	3.188 (13)	146
N6—H6*A*⋯O10^ii^	0.91	2.55	3.30 (3)	140
N3—H3*A*⋯O16^iii^	0.91	2.28	3.092 (11)	148

Symmetry codes: (i) 
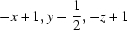
; (ii) 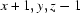; (iii) 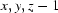.

**Fig. 1.**
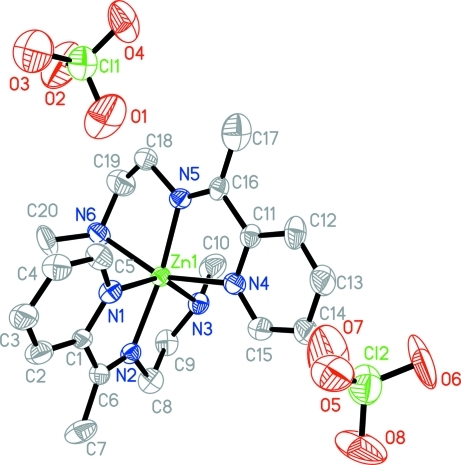
The molecular structure of the molecule 1 Zn1 complex together with its associated of the perchlorate anions, showing the atom numbering scheme. Displacement ellipsoids are drawn at the 30% probability level. H atoms are omitted for clarity.

**Fig. 2.**
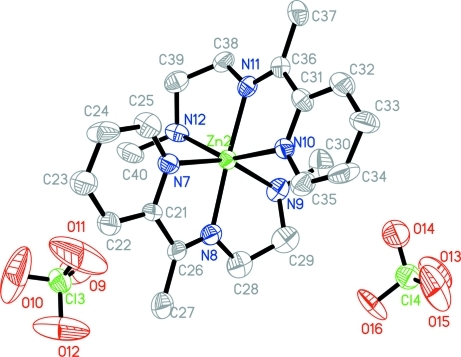
The molecular structure of the molecule 2 Zn2 complex together with its associated of the perchlorate anions, showing the atom numbering scheme. Displacement ellipsoids are drawn at the 30% probability level. H atoms are omitted for clarity.

## supplementary crystallographic information

### Comment

As part of our investigations into Schiff base complexes (Wang & Ye, 2011; Wang, 2009), we have synthesized the title compound, a new mononuclear zinc(II) complex, Fig. 1. The asymmetric unit of the complex contains two independent [Zn(C_10_H_15_N_3_)_2_]^2+^ cations and four perchlorate anions. The Zn atom in the complex is six-coordinated by two pyridine N, two imine N, and two amine N atoms from two Schiff base ligands *N*-methyl-*N*'-(1-pyridin-2-ylethylidene)ethane- 1,2-diamine, forming an octahedral geometry. The two pyridine rings in the complex cations are approximately perpendicular to each other, with the dihedral angles of 88.4 (3) and 87.9 (3)°, respectively. The *trans* angles at Zn atoms are in the range 151.3 (3)–175.1 (3)°; the other angles are in the range 72.8 (2)–113.7 (3)° (Table 1), indicating a distorted octahedral coordination. The Zn–O and Zn–N bond lengths (Table 1) are comparable with those observed in other similar zinc(II) complexes (Cai *et al.*, 2009; Yang *et al.*, 2009; Bing *et al.*, 2010; Wang *et al.*, 2010). The perchlorate anions are linked to the complex cations through N—H···O hydrogen bonds (Table 2).

### Experimental

2-Acetylpyridine (1.0 mmol, 0.121 g), *N*-methylethane-1,2-diamine (1.0 mmol, 0.074 g), and zinc perchlorate hexahydrate (1.0 mmol, 0.372 g) were dissolved in MeOH (30 ml). The mixture was stirred at room temperature for 10 min to give a clear colorless solution. After keeping the solution in air for 5 d, colorless block-shaped crystals were formed at the bottom of the vessel.

### Refinement

All H atoms were placed in geometrically idealized positions and constrained to ride on their parent atoms, with C—H distances in the range 0.93–0.97 Å, N—H distances of 0.91 Å, and with *U*_iso_(H) set at 1.2*U*_eq_(C,*N*) and 1.5*U*_eq_(methyl C).

### Crystal data

**Table:**

[Zn(C_10_H_15_N_3_)_2_](ClO_4_)_2_	*F*(000) = 1280
*M**_r_* = 618.77	*D*_x_ = 1.517 Mg m^−^^3^
Monoclinic, *P*2_1_	Mo *K*α radiation, λ = 0.71073 Å
Hall symbol: P 2yb	Cell parameters from 3298 reflections
*a* = 12.7999 (3) Å	θ = 2.5–24.6°
*b* = 15.8414 (4) Å	µ = 1.16 mm^−^^1^
*c* = 13.6869 (3) Å	*T* = 298 K
β = 102.461 (1)°	Block, colorless
*V* = 2709.89 (11) Å^3^	0.32 × 0.30 × 0.27 mm
*Z* = 4	

### Data collection

**Table:**

Bruker SMART CCD area-detector diffractometer	7055 independent reflections
Radiation source: fine-focus sealed tube	5567 reflections with *I* > 2σ(*I*)
graphite	*R*_int_ = 0.034
ω scans	θ_max_ = 23.9°, θ_min_ = 1.5°
Absorption correction: multi-scan (*SADABS*; Sheldrick, 1996)	*h* = −10→14
*T*_min_ = 0.708, *T*_max_ = 0.745	*k* = −15→18
12863 measured reflections	*l* = −14→15

### Refinement

**Table:**

Refinement on *F*^2^	Secondary atom site location: difference Fourier map
Least-squares matrix: full	Hydrogen site location: inferred from neighbouring sites
*R*[*F*^2^ > 2σ(*F*^2^)] = 0.059	H-atom parameters constrained
*wR*(*F*^2^) = 0.168	*w* = 1/[σ^2^(*F*_o_^2^) + (0.1062*P*)^2^ + 0.2728*P*] where *P* = (*F*_o_^2^ + 2*F*_c_^2^)/3
*S* = 1.04	(Δ/σ)_max_ = 0.001
7055 reflections	Δρ_max_ = 0.73 e Å^−^^3^
675 parameters	Δρ_min_ = −0.63 e Å^−^^3^
7 restraints	Absolute structure: Flack (1983), 2683 Friedel pairs
Primary atom site location: structure-invariant direct methods	Flack parameter: 0.01 (2)

### Special details

**Table:**

Geometry. All e.s.d.'s (except the e.s.d. in the dihedral angle between two l.s. planes) are estimated using the full covariance matrix. The cell e.s.d.'s are taken into account individually in the estimation of e.s.d.'s in distances, angles and torsion angles; correlations between e.s.d.'s in cell parameters are only used when they are defined by crystal symmetry. An approximate (isotropic) treatment of cell e.s.d.'s is used for estimating e.s.d.'s involving l.s. planes.
Refinement. Refinement of *F*^2^ against ALL reflections. The weighted *R*-factor *wR* and goodness of fit *S* are based on *F*^2^, conventional *R*-factors *R* are based on *F*, with *F* set to zero for negative *F*^2^. The threshold expression of *F*^2^ > σ(*F*^2^) is used only for calculating *R*-factors(gt) *etc*. and is not relevant to the choice of reflections for refinement. *R*-factors based on *F*^2^ are statistically about twice as large as those based on *F*, and *R*- factors based on ALL data will be even larger.

### Fractional atomic coordinates and isotropic or equivalent isotropic displacement parameters (Å^2^)

**Table:**

	*x*	*y*	*z*	*U*_iso_*/*U*_eq_	
Zn1	0.88515 (6)	0.30560 (5)	0.10215 (6)	0.0425 (3)	
Zn2	0.38341 (7)	0.19458 (6)	0.59030 (6)	0.0464 (3)	
Cl1	0.7463 (2)	0.0560 (2)	0.4186 (2)	0.0880 (8)	
Cl2	0.3370 (3)	0.5819 (3)	0.1430 (3)	0.1132 (11)	
Cl3	0.2472 (2)	0.1721 (2)	0.9959 (2)	0.0807 (8)	
Cl4	0.8419 (3)	0.4442 (2)	0.7056 (2)	0.0955 (9)	
O1	0.7313 (12)	0.1354 (7)	0.3791 (9)	0.182 (6)	
O2	0.8398 (9)	0.0241 (11)	0.3914 (11)	0.196 (7)	
O3	0.7689 (10)	0.0569 (9)	0.5237 (7)	0.151 (4)	
O4	0.6598 (7)	0.0056 (6)	0.3797 (9)	0.138 (4)	
O5	0.3300 (8)	0.5784 (7)	0.2452 (6)	0.122 (3)	
O6	0.2474 (7)	0.5587 (9)	0.0807 (7)	0.171 (6)	
O7	0.4172 (9)	0.5280 (11)	0.1406 (14)	0.234 (9)	
O8	0.3758 (13)	0.6601 (8)	0.1233 (8)	0.188 (7)	
O9	0.3458 (6)	0.1339 (7)	1.0385 (10)	0.149 (5)	
O10	0.1706 (10)	0.1324 (12)	1.018 (2)	0.297 (13)	
O11	0.229 (2)	0.1748 (15)	0.9013 (9)	0.295 (13)	
O12	0.2362 (12)	0.2480 (10)	1.0413 (11)	0.221 (8)	
O13	0.9530 (6)	0.4452 (10)	0.7187 (8)	0.151 (5)	
O14	0.816 (2)	0.3733 (13)	0.6554 (14)	0.339 (17)	
O15	0.8043 (11)	0.5059 (11)	0.6428 (9)	0.194 (8)	
O16	0.8055 (7)	0.4482 (7)	0.7922 (5)	0.122 (4)	
N1	0.8801 (5)	0.3548 (4)	0.2575 (5)	0.0511 (16)	
N2	0.9733 (5)	0.4188 (4)	0.1230 (5)	0.0466 (15)	
N3	0.9174 (5)	0.3229 (5)	−0.0453 (5)	0.0529 (17)	
H3A	0.8635	0.3558	−0.0797	0.064*	
N4	0.7167 (5)	0.3456 (5)	0.0521 (5)	0.0531 (17)	
N5	0.7953 (5)	0.1979 (4)	0.1151 (5)	0.0484 (15)	
N6	1.0141 (5)	0.2130 (5)	0.1570 (6)	0.0611 (19)	
H6A	1.0598	0.2203	0.1152	0.073*	
N7	0.2080 (5)	0.1975 (5)	0.5748 (5)	0.0553 (17)	
N8	0.3555 (6)	0.2852 (4)	0.6947 (5)	0.0580 (19)	
N9	0.5465 (6)	0.2398 (5)	0.6485 (6)	0.066 (2)	
H9C	0.5754	0.2065	0.7016	0.080*	
N10	0.3591 (5)	0.2709 (4)	0.4508 (5)	0.0506 (16)	
N11	0.4071 (5)	0.1126 (4)	0.4775 (5)	0.0523 (17)	
N12	0.3979 (6)	0.0720 (5)	0.6714 (5)	0.063 (2)	
H12A	0.4670	0.0675	0.7054	0.076*	
C1	0.9455 (6)	0.4204 (5)	0.2849 (5)	0.0441 (17)	
C2	0.9688 (8)	0.4491 (6)	0.3818 (7)	0.071 (3)	
H2	1.0160	0.4939	0.3991	0.085*	
C3	0.9234 (9)	0.4127 (7)	0.4535 (8)	0.078 (3)	
H3	0.9407	0.4307	0.5197	0.094*	
C4	0.8510 (8)	0.3481 (6)	0.4235 (7)	0.065 (2)	
H4	0.8159	0.3234	0.4691	0.079*	
C5	0.8314 (7)	0.3210 (6)	0.3274 (7)	0.063 (2)	
H5	0.7825	0.2774	0.3084	0.075*	
C6	0.9871 (6)	0.4594 (5)	0.2052 (7)	0.054 (2)	
C7	1.0410 (10)	0.5439 (6)	0.2197 (9)	0.084 (3)	
H7A	1.0743	0.5553	0.1645	0.127*	
H7B	1.0944	0.5438	0.2809	0.127*	
H7C	0.9889	0.5869	0.2229	0.127*	
C8	1.0080 (8)	0.4481 (7)	0.0341 (7)	0.065 (2)	
H8A	1.0772	0.4754	0.0534	0.078*	
H8B	0.9572	0.4889	−0.0015	0.078*	
C9	1.0154 (7)	0.3738 (7)	−0.0327 (7)	0.064 (2)	
H9A	1.0246	0.3935	−0.0974	0.077*	
H9B	1.0769	0.3395	−0.0032	0.077*	
C10	0.9219 (10)	0.2486 (8)	−0.1077 (9)	0.092 (3)	
H10A	0.9431	0.2654	−0.1680	0.138*	
H10B	0.8527	0.2225	−0.1242	0.138*	
H10C	0.9731	0.2092	−0.0718	0.138*	
C11	0.6467 (6)	0.2861 (6)	0.0666 (6)	0.051 (2)	
C12	0.5389 (7)	0.3028 (9)	0.0520 (7)	0.072 (3)	
H12	0.4913	0.2615	0.0634	0.087*	
C13	0.5041 (9)	0.3804 (9)	0.0210 (9)	0.086 (3)	
H13	0.4312	0.3920	0.0092	0.103*	
C14	0.5738 (8)	0.4436 (8)	0.0061 (8)	0.082 (3)	
H14	0.5502	0.4975	−0.0147	0.099*	
C15	0.6795 (7)	0.4216 (7)	0.0239 (7)	0.067 (2)	
H15	0.7286	0.4628	0.0156	0.081*	
C16	0.6942 (6)	0.2031 (6)	0.1007 (5)	0.0490 (18)	
C17	0.6187 (9)	0.1327 (9)	0.1147 (10)	0.101 (4)	
H17A	0.6394	0.0814	0.0867	0.151*	
H17B	0.5470	0.1474	0.0816	0.151*	
H17C	0.6218	0.1248	0.1848	0.151*	
C18	0.8564 (8)	0.1219 (6)	0.1499 (8)	0.066 (2)	
H18A	0.8204	0.0726	0.1165	0.080*	
H18B	0.8630	0.1152	0.2214	0.080*	
C19	0.9640 (10)	0.1310 (8)	0.1265 (11)	0.101 (4)	
H19A	1.0101	0.0865	0.1601	0.121*	
H19B	0.9575	0.1240	0.0550	0.121*	
C20	1.0787 (10)	0.2266 (10)	0.2525 (10)	0.124 (6)	
H20A	1.0374	0.2173	0.3023	0.186*	
H20B	1.1048	0.2836	0.2572	0.186*	
H20C	1.1381	0.1882	0.2633	0.186*	
C21	0.1783 (7)	0.2513 (6)	0.6400 (7)	0.059 (2)	
C22	0.0704 (9)	0.2577 (9)	0.6456 (10)	0.087 (4)	
H22	0.0490	0.2954	0.6896	0.104*	
C23	−0.0012 (10)	0.2079 (12)	0.5858 (13)	0.115 (6)	
H23	−0.0720	0.2080	0.5923	0.138*	
C24	0.0281 (8)	0.1574 (9)	0.5157 (11)	0.092 (4)	
H24	−0.0225	0.1263	0.4712	0.111*	
C25	0.1365 (7)	0.1538 (7)	0.5127 (8)	0.074 (3)	
H25	0.1580	0.1195	0.4655	0.089*	
C26	0.2624 (8)	0.2988 (6)	0.7070 (6)	0.063 (2)	
C27	0.2331 (11)	0.3632 (8)	0.7780 (9)	0.097 (4)	
H27A	0.2969	0.3904	0.8140	0.146*	
H27B	0.1980	0.3355	0.8243	0.146*	
H27C	0.1860	0.4046	0.7406	0.146*	
C28	0.4522 (9)	0.3275 (7)	0.7513 (7)	0.080 (3)	
H28A	0.4786	0.2987	0.8143	0.096*	
H28B	0.4360	0.3855	0.7655	0.096*	
C29	0.5343 (9)	0.3251 (7)	0.6884 (9)	0.086 (3)	
H29A	0.5138	0.3642	0.6331	0.103*	
H29B	0.6025	0.3435	0.7283	0.103*	
C30	0.6200 (8)	0.2383 (9)	0.5806 (11)	0.103 (4)	
H30A	0.5877	0.2663	0.5193	0.155*	
H30B	0.6356	0.1808	0.5666	0.155*	
H30C	0.6851	0.2667	0.6112	0.155*	
C31	0.3733 (6)	0.2261 (6)	0.3699 (6)	0.057 (2)	
C32	0.3646 (8)	0.2638 (8)	0.2782 (7)	0.075 (3)	
H32	0.3777	0.2329	0.2243	0.089*	
C33	0.3352 (10)	0.3511 (8)	0.2661 (8)	0.087 (3)	
H33	0.3252	0.3781	0.2044	0.105*	
C34	0.3228 (9)	0.3926 (6)	0.3509 (9)	0.086 (3)	
H34	0.3076	0.4501	0.3475	0.103*	
C35	0.3321 (8)	0.3517 (7)	0.4399 (8)	0.073 (3)	
H35	0.3191	0.3814	0.4947	0.088*	
C36	0.4025 (6)	0.1378 (5)	0.3889 (6)	0.0485 (19)	
C37	0.4197 (9)	0.0830 (7)	0.3018 (7)	0.075 (3)	
H37A	0.4947	0.0803	0.3021	0.112*	
H37B	0.3820	0.1072	0.2398	0.112*	
H37C	0.3931	0.0272	0.3087	0.112*	
C38	0.4362 (9)	0.0261 (6)	0.5132 (8)	0.071 (3)	
H38A	0.4148	−0.0139	0.4589	0.085*	
H38B	0.5130	0.0218	0.5376	0.085*	
C39	0.3813 (8)	0.0074 (6)	0.5937 (8)	0.071 (3)	
H39A	0.3052	0.0021	0.5657	0.085*	
H39B	0.4065	−0.0463	0.6237	0.085*	
C40	0.3310 (9)	0.0598 (9)	0.7436 (8)	0.090 (3)	
H40A	0.2574	0.0566	0.7093	0.135*	
H40B	0.3405	0.1064	0.7895	0.135*	
H40C	0.3511	0.0084	0.7799	0.135*	

### Atomic displacement parameters (Å^2^)

**Table:**

	*U*^11^	*U*^22^	*U*^33^	*U*^12^	*U*^13^	*U*^23^
Zn1	0.0435 (5)	0.0404 (5)	0.0437 (5)	−0.0030 (4)	0.0099 (4)	0.0000 (4)
Zn2	0.0491 (5)	0.0463 (6)	0.0440 (5)	−0.0020 (4)	0.0104 (4)	−0.0043 (4)
Cl1	0.0811 (18)	0.098 (2)	0.0861 (19)	−0.0145 (16)	0.0205 (14)	0.0061 (16)
Cl2	0.100 (2)	0.137 (3)	0.097 (2)	−0.004 (2)	0.0113 (18)	0.013 (2)
Cl3	0.0684 (15)	0.100 (2)	0.0752 (17)	0.0028 (14)	0.0183 (12)	−0.0022 (15)
Cl4	0.107 (2)	0.103 (2)	0.0740 (18)	−0.0102 (18)	0.0128 (16)	−0.0008 (18)
O1	0.305 (17)	0.086 (7)	0.129 (9)	−0.083 (9)	−0.008 (9)	0.042 (7)
O2	0.108 (8)	0.296 (19)	0.198 (13)	−0.032 (10)	0.061 (8)	−0.131 (14)
O3	0.207 (11)	0.179 (11)	0.075 (6)	0.020 (9)	0.050 (7)	0.011 (7)
O4	0.073 (5)	0.100 (7)	0.212 (11)	−0.038 (5)	−0.035 (6)	0.047 (7)
O5	0.178 (9)	0.122 (8)	0.065 (5)	−0.049 (7)	0.029 (5)	0.020 (5)
O6	0.096 (6)	0.285 (16)	0.101 (7)	−0.091 (9)	−0.045 (5)	0.016 (8)
O7	0.103 (8)	0.273 (18)	0.305 (19)	0.106 (10)	−0.001 (10)	−0.122 (16)
O8	0.310 (16)	0.148 (10)	0.090 (6)	−0.150 (11)	0.008 (8)	0.032 (7)
O9	0.069 (5)	0.127 (8)	0.233 (13)	0.028 (5)	−0.007 (6)	−0.040 (8)
O10	0.098 (9)	0.222 (18)	0.57 (4)	−0.043 (11)	0.063 (14)	0.11 (2)
O11	0.51 (3)	0.31 (2)	0.077 (8)	0.18 (2)	0.096 (13)	0.017 (11)
O12	0.223 (13)	0.208 (15)	0.182 (12)	0.129 (12)	−0.070 (10)	−0.111 (11)
O13	0.068 (5)	0.262 (14)	0.124 (8)	0.036 (7)	0.027 (5)	−0.017 (9)
O14	0.51 (3)	0.34 (2)	0.261 (19)	−0.34 (3)	0.27 (2)	−0.23 (2)
O15	0.213 (12)	0.261 (16)	0.130 (9)	0.138 (12)	0.085 (9)	0.144 (11)
O16	0.133 (7)	0.193 (10)	0.055 (4)	0.084 (7)	0.050 (4)	0.030 (5)
N1	0.057 (4)	0.052 (4)	0.047 (4)	−0.006 (3)	0.016 (3)	−0.002 (3)
N2	0.051 (4)	0.044 (4)	0.045 (4)	−0.007 (3)	0.009 (3)	−0.001 (3)
N3	0.058 (4)	0.058 (5)	0.044 (4)	0.000 (3)	0.013 (3)	−0.004 (3)
N4	0.056 (4)	0.052 (4)	0.051 (4)	0.007 (4)	0.010 (3)	0.001 (3)
N5	0.058 (4)	0.039 (4)	0.047 (4)	−0.002 (3)	0.010 (3)	0.006 (3)
N6	0.052 (4)	0.057 (5)	0.073 (5)	0.009 (3)	0.009 (3)	0.008 (4)
N7	0.052 (4)	0.059 (4)	0.056 (4)	0.000 (4)	0.014 (3)	0.008 (4)
N8	0.074 (5)	0.052 (5)	0.046 (4)	0.007 (4)	0.008 (3)	−0.005 (3)
N9	0.055 (4)	0.057 (5)	0.083 (6)	−0.007 (4)	0.005 (4)	−0.010 (4)
N10	0.058 (4)	0.048 (4)	0.049 (4)	0.000 (3)	0.017 (3)	−0.002 (3)
N11	0.057 (4)	0.042 (4)	0.057 (5)	0.000 (3)	0.010 (3)	−0.009 (3)
N12	0.066 (4)	0.068 (5)	0.055 (4)	−0.010 (4)	0.011 (3)	0.014 (4)
C1	0.052 (4)	0.041 (4)	0.038 (4)	0.003 (4)	0.008 (3)	−0.003 (3)
C2	0.095 (7)	0.055 (6)	0.065 (6)	0.002 (5)	0.021 (5)	−0.010 (5)
C3	0.112 (8)	0.071 (7)	0.052 (6)	0.016 (6)	0.020 (6)	−0.007 (5)
C4	0.081 (6)	0.066 (6)	0.058 (6)	0.004 (5)	0.033 (5)	0.003 (5)
C5	0.073 (6)	0.063 (6)	0.056 (5)	0.002 (5)	0.021 (4)	0.020 (5)
C6	0.044 (4)	0.047 (5)	0.065 (5)	0.000 (4)	0.001 (4)	−0.004 (4)
C7	0.124 (9)	0.053 (6)	0.084 (7)	−0.027 (6)	0.038 (7)	−0.008 (5)
C8	0.070 (6)	0.071 (6)	0.060 (5)	−0.011 (5)	0.028 (5)	0.007 (5)
C9	0.053 (5)	0.088 (7)	0.057 (5)	0.000 (5)	0.023 (4)	−0.001 (5)
C10	0.120 (9)	0.089 (8)	0.068 (7)	−0.007 (7)	0.021 (6)	−0.015 (6)
C11	0.049 (5)	0.062 (6)	0.042 (4)	−0.001 (4)	0.011 (3)	−0.006 (4)
C12	0.045 (5)	0.113 (9)	0.059 (5)	−0.010 (6)	0.011 (4)	−0.009 (6)
C13	0.051 (6)	0.110 (10)	0.094 (8)	0.013 (7)	0.012 (5)	−0.026 (7)
C14	0.059 (6)	0.087 (8)	0.094 (8)	0.018 (6)	0.000 (5)	−0.024 (7)
C15	0.064 (6)	0.058 (6)	0.078 (6)	0.006 (5)	0.011 (5)	−0.005 (5)
C16	0.053 (5)	0.055 (5)	0.037 (4)	−0.007 (4)	0.007 (3)	−0.002 (4)
C17	0.082 (7)	0.117 (10)	0.104 (9)	−0.042 (7)	0.022 (6)	0.018 (8)
C18	0.077 (6)	0.048 (5)	0.067 (6)	0.002 (4)	0.001 (5)	0.006 (4)
C19	0.102 (7)	0.077 (6)	0.117 (8)	0.010 (6)	0.008 (6)	0.011 (6)
C20	0.103 (9)	0.144 (13)	0.097 (9)	0.064 (9)	−0.041 (7)	−0.014 (9)
C21	0.064 (5)	0.062 (6)	0.059 (6)	0.018 (5)	0.028 (5)	0.024 (5)
C22	0.057 (6)	0.107 (9)	0.105 (9)	0.027 (6)	0.036 (6)	0.047 (8)
C23	0.062 (8)	0.146 (14)	0.149 (13)	0.044 (9)	0.046 (9)	0.086 (12)
C24	0.047 (6)	0.107 (10)	0.111 (9)	0.002 (6)	−0.008 (6)	0.060 (9)
C25	0.051 (5)	0.100 (8)	0.065 (6)	−0.009 (5)	−0.001 (4)	0.009 (6)
C26	0.090 (7)	0.057 (6)	0.048 (5)	0.024 (5)	0.027 (5)	0.007 (5)
C27	0.149 (11)	0.084 (8)	0.072 (7)	0.035 (7)	0.054 (7)	−0.004 (6)
C28	0.101 (8)	0.070 (7)	0.062 (6)	−0.012 (6)	0.001 (5)	−0.030 (5)
C29	0.076 (7)	0.074 (8)	0.097 (8)	−0.021 (6)	−0.002 (6)	−0.016 (6)
C30	0.061 (6)	0.118 (10)	0.140 (11)	−0.039 (7)	0.043 (7)	−0.031 (9)
C31	0.050 (5)	0.072 (6)	0.052 (5)	−0.010 (4)	0.015 (4)	−0.002 (4)
C32	0.093 (7)	0.083 (7)	0.049 (5)	0.002 (6)	0.015 (5)	0.000 (5)
C33	0.130 (10)	0.076 (8)	0.059 (6)	−0.003 (7)	0.027 (6)	0.015 (6)
C34	0.120 (9)	0.041 (6)	0.089 (8)	−0.002 (5)	0.009 (7)	0.016 (6)
C35	0.092 (7)	0.058 (7)	0.068 (6)	0.007 (5)	0.015 (5)	−0.002 (5)
C36	0.045 (4)	0.052 (5)	0.048 (5)	−0.002 (4)	0.008 (3)	−0.006 (4)
C37	0.098 (7)	0.074 (7)	0.059 (6)	0.005 (6)	0.030 (5)	−0.015 (5)
C38	0.092 (7)	0.048 (5)	0.074 (6)	0.015 (5)	0.020 (5)	0.002 (5)
C39	0.081 (7)	0.048 (6)	0.080 (7)	−0.006 (5)	0.008 (5)	−0.006 (5)
C40	0.094 (7)	0.104 (9)	0.077 (7)	−0.008 (7)	0.028 (6)	0.030 (7)

### Geometric parameters (Å, °)

**Table:**

Zn1—N5	2.087 (6)	C8—H8A	0.9700
Zn1—N2	2.105 (6)	C8—H8B	0.9700
Zn1—N3	2.163 (6)	C9—H9A	0.9700
Zn1—N4	2.210 (7)	C9—H9B	0.9700
Zn1—N6	2.215 (7)	C10—H10A	0.9600
Zn1—N1	2.278 (6)	C10—H10B	0.9600
Zn2—N11	2.090 (7)	C10—H10C	0.9600
Zn2—N8	2.109 (7)	C11—C12	1.378 (12)
Zn2—N9	2.188 (7)	C11—C16	1.481 (12)
Zn2—N7	2.209 (6)	C12—C13	1.344 (18)
Zn2—N12	2.224 (7)	C12—H12	0.9300
Zn2—N10	2.225 (7)	C13—C14	1.385 (17)
Cl1—O1	1.366 (10)	C13—H13	0.9300
Cl1—O4	1.375 (8)	C14—C15	1.368 (13)
Cl1—O3	1.406 (10)	C14—H14	0.9300
Cl1—O2	1.421 (11)	C15—H15	0.9300
Cl2—O6	1.324 (8)	C16—C17	1.514 (13)
Cl2—O7	1.342 (10)	C17—H17A	0.9600
Cl2—O8	1.384 (10)	C17—H17B	0.9600
Cl2—O5	1.421 (8)	C17—H17C	0.9600
Cl3—O10	1.256 (14)	C18—C19	1.487 (16)
Cl3—O11	1.267 (12)	C18—H18A	0.9700
Cl3—O12	1.374 (12)	C18—H18B	0.9700
Cl3—O9	1.408 (9)	C19—H19A	0.9700
Cl4—O14	1.321 (13)	C19—H19B	0.9700
Cl4—O15	1.322 (10)	C20—H20A	0.9600
Cl4—O16	1.365 (7)	C20—H20B	0.9600
Cl4—O13	1.394 (8)	C20—H20C	0.9600
N1—C1	1.338 (10)	C21—C22	1.404 (13)
N1—C5	1.359 (10)	C21—C26	1.463 (14)
N2—C6	1.275 (10)	C22—C23	1.35 (2)
N2—C8	1.458 (10)	C22—H22	0.9300
N3—C10	1.462 (13)	C23—C24	1.36 (2)
N3—C9	1.470 (11)	C23—H23	0.9300
N3—H3A	0.9100	C24—C25	1.398 (14)
N4—C15	1.321 (12)	C24—H24	0.9300
N4—C11	1.344 (10)	C25—H25	0.9300
N5—C16	1.269 (9)	C26—C27	1.509 (13)
N5—C18	1.457 (11)	C27—H27A	0.9600
N6—C20	1.405 (13)	C27—H27B	0.9600
N6—C19	1.468 (15)	C27—H27C	0.9600
N6—H6A	0.9100	C28—C29	1.496 (15)
N7—C25	1.306 (12)	C28—H28A	0.9700
N7—C21	1.346 (12)	C28—H28B	0.9700
N8—C26	1.258 (11)	C29—H29A	0.9700
N8—C28	1.472 (12)	C29—H29B	0.9700
N9—C30	1.459 (12)	C30—H30A	0.9600
N9—C29	1.478 (13)	C30—H30B	0.9600
N9—H9C	0.9100	C30—H30C	0.9600
N10—C35	1.326 (12)	C31—C32	1.373 (13)
N10—C31	1.359 (11)	C31—C36	1.456 (13)
N11—C36	1.265 (11)	C32—C33	1.434 (15)
N11—C38	1.476 (12)	C32—H32	0.9300
N12—C40	1.454 (11)	C33—C34	1.373 (15)
N12—C39	1.457 (12)	C33—H33	0.9300
N12—H12A	0.9100	C34—C35	1.362 (14)
C1—C2	1.372 (12)	C34—H34	0.9300
C1—C6	1.451 (11)	C35—H35	0.9300
C2—C3	1.371 (14)	C36—C37	1.529 (12)
C2—H2	0.9300	C37—H37A	0.9600
C3—C4	1.382 (14)	C37—H37B	0.9600
C3—H3	0.9300	C37—H37C	0.9600
C4—C5	1.355 (13)	C38—C39	1.460 (13)
C4—H4	0.9300	C38—H38A	0.9700
C5—H5	0.9300	C38—H38B	0.9700
C6—C7	1.500 (13)	C39—H39A	0.9700
C7—H7A	0.9600	C39—H39B	0.9700
C7—H7B	0.9600	C40—H40A	0.9600
C7—H7C	0.9600	C40—H40B	0.9600
C8—C9	1.506 (14)	C40—H40C	0.9600
			
N5—Zn1—N2	167.0 (2)	N3—C9—H9B	109.7
N5—Zn1—N3	113.7 (3)	C8—C9—H9B	109.7
N2—Zn1—N3	79.3 (3)	H9A—C9—H9B	108.2
N5—Zn1—N4	75.0 (3)	N3—C10—H10A	109.5
N2—Zn1—N4	104.9 (3)	N3—C10—H10B	109.5
N3—Zn1—N4	92.5 (3)	H10A—C10—H10B	109.5
N5—Zn1—N6	79.2 (3)	N3—C10—H10C	109.5
N2—Zn1—N6	100.4 (3)	H10A—C10—H10C	109.5
N3—Zn1—N6	97.0 (3)	H10B—C10—H10C	109.5
N4—Zn1—N6	154.3 (3)	N4—C11—C12	121.5 (9)
N5—Zn1—N1	94.2 (2)	N4—C11—C16	115.4 (7)
N2—Zn1—N1	72.8 (2)	C12—C11—C16	123.1 (9)
N3—Zn1—N1	151.3 (3)	C13—C12—C11	118.4 (11)
N4—Zn1—N1	88.2 (2)	C13—C12—H12	120.8
N6—Zn1—N1	94.6 (3)	C11—C12—H12	120.8
N11—Zn2—N8	175.1 (3)	C12—C13—C14	121.8 (10)
N11—Zn2—N9	101.1 (3)	C12—C13—H13	119.1
N8—Zn2—N9	79.5 (3)	C14—C13—H13	119.1
N11—Zn2—N7	104.4 (3)	C15—C14—C13	115.7 (11)
N8—Zn2—N7	74.6 (3)	C15—C14—H14	122.2
N9—Zn2—N7	153.9 (3)	C13—C14—H14	122.2
N11—Zn2—N12	79.4 (3)	N4—C15—C14	124.4 (10)
N8—Zn2—N12	105.3 (3)	N4—C15—H15	117.8
N9—Zn2—N12	97.7 (3)	C14—C15—H15	117.8
N7—Zn2—N12	92.3 (3)	N5—C16—C11	116.2 (7)
N11—Zn2—N10	73.7 (3)	N5—C16—C17	126.2 (9)
N8—Zn2—N10	101.5 (3)	C11—C16—C17	117.6 (8)
N9—Zn2—N10	94.6 (3)	C16—C17—H17A	109.5
N7—Zn2—N10	87.4 (2)	C16—C17—H17B	109.5
N12—Zn2—N10	152.1 (3)	H17A—C17—H17B	109.5
O1—Cl1—O4	110.5 (7)	C16—C17—H17C	109.5
O1—Cl1—O3	112.0 (8)	H17A—C17—H17C	109.5
O4—Cl1—O3	111.9 (7)	H17B—C17—H17C	109.5
O1—Cl1—O2	106.1 (11)	N5—C18—C19	107.8 (8)
O4—Cl1—O2	110.2 (8)	N5—C18—H18A	110.1
O3—Cl1—O2	105.8 (8)	C19—C18—H18A	110.1
O6—Cl2—O7	111.6 (10)	N5—C18—H18B	110.1
O6—Cl2—O8	114.9 (9)	C19—C18—H18B	110.1
O7—Cl2—O8	104.8 (11)	H18A—C18—H18B	108.5
O6—Cl2—O5	113.2 (7)	N6—C19—C18	113.1 (10)
O7—Cl2—O5	102.2 (10)	N6—C19—H19A	109.0
O8—Cl2—O5	109.2 (7)	C18—C19—H19A	109.0
O10—Cl3—O11	106.6 (17)	N6—C19—H19B	109.0
O10—Cl3—O12	99.6 (13)	C18—C19—H19B	109.0
O11—Cl3—O12	114.8 (12)	H19A—C19—H19B	107.8
O10—Cl3—O9	111.2 (10)	N6—C20—H20A	109.5
O11—Cl3—O9	112.7 (10)	N6—C20—H20B	109.5
O12—Cl3—O9	111.0 (7)	H20A—C20—H20B	109.5
O14—Cl4—O15	106.0 (14)	N6—C20—H20C	109.5
O14—Cl4—O16	113.5 (8)	H20A—C20—H20C	109.5
O15—Cl4—O16	112.7 (7)	H20B—C20—H20C	109.5
O14—Cl4—O13	101.9 (12)	N7—C21—C22	120.3 (10)
O15—Cl4—O13	107.1 (9)	N7—C21—C26	117.8 (7)
O16—Cl4—O13	114.7 (6)	C22—C21—C26	121.8 (10)
C1—N1—C5	117.6 (7)	C23—C22—C21	118.2 (13)
C1—N1—Zn1	112.7 (5)	C23—C22—H22	120.9
C5—N1—Zn1	129.2 (6)	C21—C22—H22	120.9
C6—N2—C8	124.5 (7)	C22—C23—C24	121.2 (12)
C6—N2—Zn1	121.2 (5)	C22—C23—H23	119.4
C8—N2—Zn1	114.1 (5)	C24—C23—H23	119.4
C10—N3—C9	111.7 (7)	C23—C24—C25	118.1 (13)
C10—N3—Zn1	118.8 (6)	C23—C24—H24	120.9
C9—N3—Zn1	106.8 (5)	C25—C24—H24	120.9
C10—N3—H3A	106.2	N7—C25—C24	121.3 (12)
C9—N3—H3A	106.2	N7—C25—H25	119.4
Zn1—N3—H3A	106.2	C24—C25—H25	119.4
C15—N4—C11	118.2 (7)	N8—C26—C21	114.6 (8)
C15—N4—Zn1	128.1 (6)	N8—C26—C27	125.2 (10)
C11—N4—Zn1	113.0 (5)	C21—C26—C27	120.0 (9)
C16—N5—C18	124.1 (7)	C26—C27—H27A	109.5
C16—N5—Zn1	119.8 (6)	C26—C27—H27B	109.5
C18—N5—Zn1	115.9 (5)	H27A—C27—H27B	109.5
C20—N6—C19	122.3 (10)	C26—C27—H27C	109.5
C20—N6—Zn1	117.4 (7)	H27A—C27—H27C	109.5
C19—N6—Zn1	104.0 (6)	H27B—C27—H27C	109.5
C20—N6—H6A	103.6	N8—C28—C29	107.5 (8)
C19—N6—H6A	103.6	N8—C28—H28A	110.2
Zn1—N6—H6A	103.6	C29—C28—H28A	110.2
C25—N7—C21	120.6 (8)	N8—C28—H28B	110.2
C25—N7—Zn2	127.3 (7)	C29—C28—H28B	110.2
C21—N7—Zn2	112.1 (6)	H28A—C28—H28B	108.5
C26—N8—C28	124.3 (8)	N9—C29—C28	112.3 (9)
C26—N8—Zn2	120.8 (6)	N9—C29—H29A	109.1
C28—N8—Zn2	114.9 (6)	C28—C29—H29A	109.1
C30—N9—C29	112.7 (9)	N9—C29—H29B	109.1
C30—N9—Zn2	117.3 (7)	C28—C29—H29B	109.1
C29—N9—Zn2	105.4 (6)	H29A—C29—H29B	107.9
C30—N9—H9C	107.0	N9—C30—H30A	109.5
C29—N9—H9C	107.0	N9—C30—H30B	109.5
Zn2—N9—H9C	107.0	H30A—C30—H30B	109.5
C35—N10—C31	119.4 (8)	N9—C30—H30C	109.5
C35—N10—Zn2	127.3 (7)	H30A—C30—H30C	109.5
C31—N10—Zn2	113.3 (6)	H30B—C30—H30C	109.5
C36—N11—C38	124.6 (8)	N10—C31—C32	121.2 (9)
C36—N11—Zn2	121.8 (6)	N10—C31—C36	115.5 (8)
C38—N11—Zn2	113.5 (6)	C32—C31—C36	123.2 (9)
C40—N12—C39	112.9 (8)	C31—C32—C33	119.5 (10)
C40—N12—Zn2	117.2 (7)	C31—C32—H32	120.3
C39—N12—Zn2	105.4 (5)	C33—C32—H32	120.3
C40—N12—H12A	106.9	C34—C33—C32	116.1 (10)
C39—N12—H12A	106.9	C34—C33—H33	122.0
Zn2—N12—H12A	106.9	C32—C33—H33	122.0
N1—C1—C2	121.4 (8)	C35—C34—C33	121.7 (10)
N1—C1—C6	115.4 (7)	C35—C34—H34	119.2
C2—C1—C6	123.2 (8)	C33—C34—H34	119.2
C3—C2—C1	121.1 (10)	N10—C35—C34	122.0 (10)
C3—C2—H2	119.5	N10—C35—H35	119.0
C1—C2—H2	119.5	C34—C35—H35	119.0
C2—C3—C4	117.4 (9)	N11—C36—C31	115.5 (8)
C2—C3—H3	121.3	N11—C36—C37	125.6 (8)
C4—C3—H3	121.3	C31—C36—C37	118.7 (8)
C5—C4—C3	119.7 (9)	C36—C37—H37A	109.5
C5—C4—H4	120.2	C36—C37—H37B	109.5
C3—C4—H4	120.2	H37A—C37—H37B	109.5
C4—C5—N1	122.8 (9)	C36—C37—H37C	109.5
C4—C5—H5	118.6	H37A—C37—H37C	109.5
N1—C5—H5	118.6	H37B—C37—H37C	109.5
N2—C6—C1	116.5 (7)	C39—C38—N11	108.1 (8)
N2—C6—C7	122.6 (8)	C39—C38—H38A	110.1
C1—C6—C7	120.9 (8)	N11—C38—H38A	110.1
C6—C7—H7A	109.5	C39—C38—H38B	110.1
C6—C7—H7B	109.5	N11—C38—H38B	110.1
H7A—C7—H7B	109.5	H38A—C38—H38B	108.4
C6—C7—H7C	109.5	N12—C39—C38	113.0 (8)
H7A—C7—H7C	109.5	N12—C39—H39A	109.0
H7B—C7—H7C	109.5	C38—C39—H39A	109.0
N2—C8—C9	109.1 (8)	N12—C39—H39B	109.0
N2—C8—H8A	109.9	C38—C39—H39B	109.0
C9—C8—H8A	109.9	H39A—C39—H39B	107.8
N2—C8—H8B	109.9	N12—C40—H40A	109.5
C9—C8—H8B	109.9	N12—C40—H40B	109.5
H8A—C8—H8B	108.3	H40A—C40—H40B	109.5
N3—C9—C8	109.6 (6)	N12—C40—H40C	109.5
N3—C9—H9A	109.7	H40A—C40—H40C	109.5
C8—C9—H9A	109.7	H40B—C40—H40C	109.5

### Hydrogen-bond geometry (Å, °)

**Table:**

*D*—H···*A*	*D*—H	H···*A*	*D*···*A*	*D*—H···*A*
N12—H12A···O5^i^	0.91	2.55	3.425 (13)	163.
N12—H12A···O7^i^	0.91	2.38	3.175 (17)	146.
N9—H9C···O8^i^	0.91	2.45	3.315 (14)	158.
N9—H9C···O5^i^	0.91	2.39	3.188 (13)	146.
N6—H6A···O10^ii^	0.91	2.55	3.30 (3)	140.
N3—H3A···O16^iii^	0.91	2.28	3.092 (11)	148.

Symmetry codes: (i) −*x*+1, *y*−1/2, −*z*+1; (ii) *x*+1, *y*, *z*−1; (iii) *x*, *y*, *z*−1.
